# Synthetic biology toolkit for engineering *Cupriviadus necator* H16 as a platform for CO_2_ valorization

**DOI:** 10.1186/s13068-021-02063-0

**Published:** 2021-11-04

**Authors:** Haojie Pan, Jia Wang, Haoliang Wu, Zhongjian Li, Jiazhang Lian

**Affiliations:** 1 Key Laboratory of Biomass Chemical Engineering of Ministry of Education, College of Chemical and Biological Engineering, Zhejiang University, Hangzhou, 310027 China; 2 Hangzhou Global Scientific and Technological Innovation Center, Zhejiang University, Hangzhou, 310027 China

**Keywords:** *Cupriviadus necator* H16, *Ralstonia eutropha* H16, Synthetic biology, Metabolic engineering, CO_2_ conversion, Biomanufacturing

## Abstract

**Background:**

CO_2_ valorization is one of the effective methods to solve current environmental and energy problems, in which microbial electrosynthesis (MES) system has proved feasible and efficient. *Cupriviadus necator* (*Ralstonia eutropha*) H16, a model chemolithoautotroph, is a microbe of choice for CO_2_ conversion, especially with the ability to be employed in MES due to the presence of genes encoding [NiFe]-hydrogenases and all the Calvin–Benson–Basham cycle enzymes. The CO_2_ valorization strategy will make sense because the required hydrogen can be produced from renewable electricity independently of fossil fuels.

**Main body:**

In this review, synthetic biology toolkit for *C. necator* H16, including genetic engineering vectors, heterologous gene expression elements, platform strain and genome engineering, and transformation strategies, is firstly summarized. Then, the review discusses how to apply these tools to make *C. necator* H16 an efficient cell factory for converting CO_2_ to value-added products, with the examples of alcohols, fatty acids, and terpenoids. The review is concluded with the limitation of current genetic tools and perspectives on the development of more efficient and convenient methods as well as the extensive applications of *C. necator* H16.

**Conclusions:**

Great progress has been made on genetic engineering toolkit and synthetic biology applications of *C. necator* H16. Nevertheless, more efforts are expected in the near future to engineer *C. necator* H16 as efficient cell factories for the conversion of CO_2_ to value-added products.

## Background

With increasing concerns on climate change and sustainability, new concepts such as “Circular Economies” and “Carbon Neutrality” have been proposed to call for the production of chemicals and biofuels from renewable feedstocks [1, 2]. However, corn-based biofuel production has triggered a fierce debate on “Food versus Fuel” [3] and the second-generation biofuels from lignocellulose biomass still suffer from low efficiency and high cost [4]. Alternatively, CO_2_ is generally considered as the third-generation feedstock for biofuels [5]. The realization of CO_2_ recycling will be an effective way to address current challenges in energy, resource, and environment. Currently, many efforts have been devoted to establishing efficient CO_2_ conversion systems. On one hand, chemists have been designing and engineering new catalysts for converting CO_2_ to fuels such as CO, CH_4_, and CH_3_OH with high energy efficiency, although the product portfolio is expected to be further expanded [6, 7]. On the other hand, via metabolic engineering of photosynthetic microorganisms such as cyanobacteria, biologists have realized the direct conversion of CO_2_ to various value-added products [8]. In recent years, microbial electrosynthesis (MES) system, coupling electrocatalysis with microorganisms, is a feasible strategy that can be more efficient than natural photosynthesis for converting CO_2_ to complex and high-value products [9, 10]. In MES, CO_2_ is fixed and reduced by microbial cells and the required redox equivalents, such as formate, H_2_, and electrons, are provided by electrochemical reactions [11, 12].

*Cupriviadus necator* H16, also known as *Hydrogenomonas* H16, *Alcaligenes eutrophus* H16, *Wautersia eutropha* H16, and *Ralstonia eutropha* H16, is a Gram-negative betaproteobacterium discovered 60 years ago [13–16]. It has gained intensive research interests in recent years due to its capability of CO_2_ fixation and conversion, especially in MES [10, 11, 17–26]. *C. necator* represents a model facultative chemolithoautotroph that can utilize fructose, gluconate, various organic acid, and CO_2_ as carbon sources. While carbohydrates are metabolized via the Entner–Doudoroff (ED) Pathway with 2-keto-3-deoxy-6-phosphogluconate aldolase (Eda) as the key enzyme, *C. necator* H16 also embraces all the necessary genes of the entire Calvin–Benson–Basham (CBB) cycle. Besides, two oxygen-tolerant [NiFe]-hydrogenases enable *C. necator* H16 to power the reduction of CO_2_ by hydrogen [27–30]. Another research hotspot about *C. necator* H16 is polyhydroxyalkanoate (PHA) production, which is a kind of biodegradable materials with high thermoplasticity, elasticity, and biocompatibility. PHA biosynthesis has been extensively reviewed and will not be discussed here [31, 32]. While the carbon source utilization range [33] and the product scopes [34] of *C. necator* H16 have been reviewed elsewhere, the review is mainly focused on the development of synthetic biology tools. Complete genomic information [35–37] has laid the foundation for the development of these tools, and transcriptomics, proteomics, and metabolomics studies have provided the guidance for metabolic engineering [38–44].

**Fig. 1:**
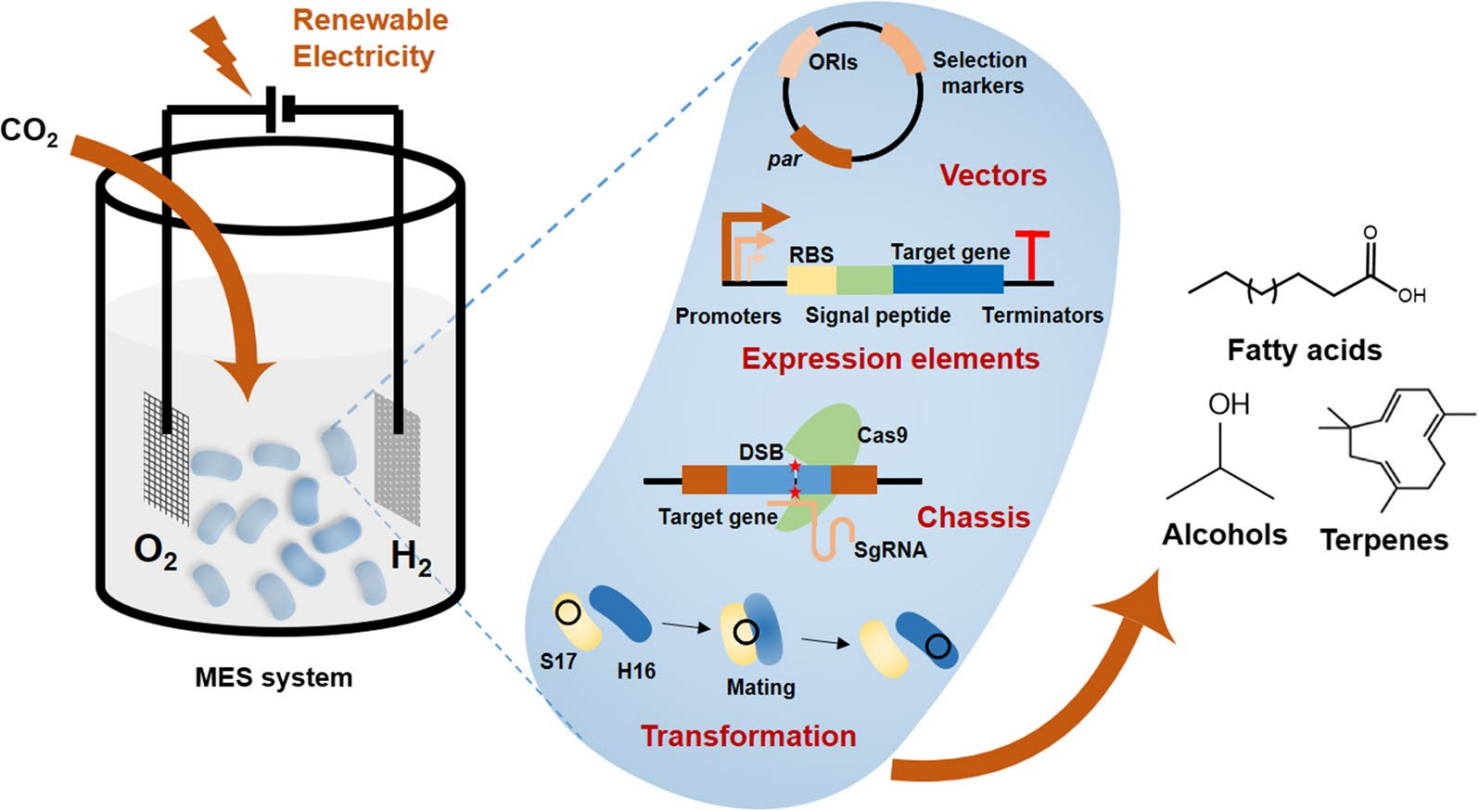
Overview of synthetic biology toolkit for *C. necator* H16 and microbial electrosynthesis (MES) system. A well-established MES system requires good coupling of *C. necator* with the electrolysis system and efficient CO_2_ conversion to value-added products. ORI: origin of replication

In this review, synthetic biology toolkit available for *C*. *necator* H16 is firstly summarized, whose applications in engineering *C. necator* H16 as efficient cell factories for converting CO_2_ to value-added products is followed for discussion (Fig. 1). The review is concluded with the limitations of current genetic tools and perspectives on extensive applications of *C. necator* H16.

## Genetic engineering vectors

Vectors, based on either episomal plasmids or chromosomal insertion, introduce and stabilize heterologous genes in recipient cells. Up to now, various vectors have been established in *C. necator*, including autonomous replication elements for episomal plasmids and integration sites for chromosomal insertion [45–47]. The former is easy to control the copy numbers but suffers from serious plasmid loss, while the latter has high stability but low copy numbers. Therefore, how to choose appropriate expression vectors according to the target products is a worth-thinking and open-to-discussion question. Luckily, systems for plasmid stability and maintenance developed in the past few years offer more options for metabolic engineering [46, 48–52].

### Episomal plasmids

According to different replication mechanisms, plasmids can be assigned to different incompatibility groups. Most of incompatibility groups commonly used in Gram-negative bacteria are adopted successfully in *C. necator*: IncQ-group (e.g., RSF1010 [53, 54] and pJRD derived from RSF1010 [55]), IncP-group (e.g., RP4 (RK2) [56]), IncW-group (e.g., pSa [46]), and pBBR1 [57]. pBBR1 is the most commonly used in *C. necator*. In addition, the plasmid, pCUP3, harboring the plasmid partition and replication region of the mega-plasmid pMOL28 from *Ralstonia metallidurans* CH43, shows effectivity in *C. necator* H16 and compatibility with the native plasmid, pHG1 [48]. The co-existence of plasmids from different incompatibility groups in one cell was found to be feasible. Li et al. achieved autotrophic production of fatty acids using plasmids based on pBBR1 and RP4 [58] and Claassens et al. replaced the CBB cycle with the reductive glycine pathway using plasmids based on RP4 and RSF1010 [59].

A properly regulated replication system, including cis-region (e.g., origin of replication, ORI) and trans-region (e.g., replication proteins), largely determined the copy numbers and stability during cell division [45]. Plasmids with different ORIs and accordingly different copy numbers are listed in Table 1. It is noteworthy that copy numbers of the RP4-based plasmids (e.g., pCM) are regulated by the *trfA* gene [60] and site-directed mutagenesis of *trfA* could increase the plasmid copy numbers to even higher than 40 per cell [47]. As for the plasmid stability, no comparative study has been conducted under the same condition. Generally, all the episomal plasmids suffer from segregational instability. Taking the RSF1010-based plasmid for example, even in the presence of chloramphenicol, the percentage of plasmid-bearing cells was reduced to 10% after 70 h of fermentation [54]. As for the pBBR1-based plasmid, about 95% and 80% were lost in minimal medium (MM) and LB after four subcultures without antibiotic pressure, respectively [61]. RP4-derived plasmids are considered as the most stable in *C. necator*. However, only about 70% of the cells carrying RP4-based plasmid cultured in the tetracycline-free medium were tetracycline resistant compared to those cultured with tetracycline after 24 h of fermentation [49].

**Table 1:** Genetic engineering vectors commonly used in *C. necator*

Vectors	Description	Reference
**Episomal plasmids**		
pCM	RP4 ORI, low copy, ~ 8	[47]
pCM271	RP4 ORI, mutation in *trfA*, medium copy, ~ 40	[47]
pBBR1	medium copy, ~ 40	[57]
pKT230	RSF1010 ORI, medium copy, ~ 10	[47]
pSa	low copy, 2 ~ 3	[46]
pCUP3	pMOL28 ORI, low copy, 1	[48]
**Integrative plasmids**		
pJQPPCm	*phaP* (Phasin) as integration locus	[53, 54]
pJV7	*phaC1* (PHA synthase) as integration locus	[51]
pCB42	*phaB1* (acetoacetyl-CoA reductase) as integration locus	[66]
pLH63	*phaB2C2* as integration locus	[26]
pEX100T	*ldh* (L-lactate dehydrogenase) as integration locus	[52]
pLO1	*norR2A2B2* (NO reductase) as integration locus	[49, 68]

### Elements for plasmid stability and maintenance

A common strategy for plasmid maintenance is to keep the selection pressure, such as the addition of antibiotics to the medium. Three antibiotics have been found to work as screening pressure in *C. necator* (Table 2): kanamycin, tetracycline, and chloramphenicol. The working concentration to some extent is dependent on the culture conditions and drug manufacturers. However, antibiotic supplementation is not a good choice for large-scale fermentation due to its financial expenses and negative environmental impact [62]. In addition, antibiotic selection suffers from low stability. The study of Voss et al. showed that 38% of the tetracycline-resistant and 62% of the kanamycin-resistant cells lost their plasmids during cultivation [52]. To address the limitation of antibiotics, two additional strategies have been employed: toxin/anti-toxin system and metabolism-based plasmid addiction system.

**Table 2:** Screening markers available in *C. necator*

Marker	Description	Reference
**Resistance marker**		
*Neo*^*R*^/*Kan*^*R*^	Resistant to kanamycin; 200–350 μg/mL	[47]
*Tc*^*R*^	Resistant to tetracycline; 10–25 μg/mL	[47]
*Cm*^*R*^	Resistant to chloramphenicol; 25–50 μg/mL	[47]
**Nutrition-deficient complementary marker**		
*hoxABCJ*	*C. necato*rΔ*hoxA*; transcription regulator of two [NiFe]-Hydrogenases	[49]
*eda*	*C. necato*rΔ*eda*; key enzyme of ED pathway	[52]
*xfp*	*C. necato*rΔ*eda*; key enzyme of the alternative phosphoketolase-dependent pathway	[50]
*proC*	*C. necato*r Δ*proC*; indispensable gene for proline biosynthesis	[51]
**Counter-selectable marker**		
*sacB*	Lethal in sucrose-containing medium	[114]

The antibiotic ranges to some extent is dependent on the culture conditions and drug manufacturers

Toxin/anti-toxin is a mechanism to regulate cell growth and death under various stress conditions in bacteria and archaea. These toxins are highly stable and may inhibit cell growth or even cause cell death by blocking essential cellular processes, whereas cognate anti-toxins can relieve such toxicity but continuous expression is required due to low stability. Thus, once the plasmid bearing the toxin/anti-toxin operon was lost, previously expressed and stably retained toxins would kill or inhibit the growth of the plasmid-free cells [63]. The pMOL28-derived parABS28 is such a system that shows decent performance for plasmid partition and maintenance. Sato et al. showed nearly no plasmid loss due to this post-segregational killing system [48]. RP4 partitioning system from natural plasmid RP4 (RK2) also contains toxin/anti-toxin system (i.e., parDE) [64]. Gruber et al. combined RP4 partition sequence with different ORIs (PR4, RSF1010, pBBR1, and pSa) and obtained highly stable plasmids independent of the replication system [46]. The studies done by Sydow et al. [61] and Krieg et al. [19] further demonstrated the effectiveness of this strategy.

The metabolism-based plasmid addiction system is comparable to the nutrition-deficient complementary system in yeast. As mentioned above, *eda* encodes a key enzyme for carbohydrate metabolism in *C. necator*. Voss et al. knocked out *eda* gene in the host and constructed pBBR1MCS-2 bearing complementary *eda* to produce cyanophycin. 93% of the cells maintained the plasmids without antibiotic selection and the production was at least fivefold higher [52]. The phosphoketolase-dependent pathway (the key gene is *xfp*) is an alternative way to restore the capability of fructose utilization. Fleige et al. heterologously expressed *xfp* gene from *Bifidobacterium animalis* on the plasmids in the *eda*-deficient *C. necator* strain to obtain a stable production platform [50]. A similar system was established based on the pyrroline-5-carboxylate reductase gene (*proC*), an indispensable gene for proline biosynthesis, which was useful in MM where proline is not supplemented [51]. Lutte et al. complemented the deficiency in the native hydrogenase transcription factor *hoxA* with *hoxABCJ* from *Alcaligenes sp.* M50 on the plasmids to construct a novel addiction system under lithoautotrophic conditions, which showed no plasmid loss and higher production of cyanophycin [49] (Table 2).

### Genome integration vectors

As high copy number plasmids usually exert metabolic burdens on cell growth, integration into the chromosome is preferred. Target gene expression cassettes are generally cloned between two ~ 500 bp homologous arms and integrated into specific genome sites [49, 52, 65]. Commonly used integration sites in *C. necator* are listed in Table 1. Noteworthy, most of integration sites are related to the polyhydroxybutyrate (PHB) biosynthetic pathway, which is one of the most thoroughly studied metabolic pathways and whose disruption can easily redirect carbon flux to produce the target products. Srinivasan et al. integrated P_*phaP*_-*OPH* into the *phaP* site and achieved soluble and functional expression of organophosphohydrolase, a protein prone to form inclusion body in *Escherichia coli* [53]. Budde et al. replaced *phaB1* and *phaC1* with *phaB* and *phaC* homologs, respectively, to investigate their functions [51, 66]. Li et al. integrated part of the isobutanol and 3-methyl-1-butanol biosynthetic pathway into the *phaB2C2* site, with the remaining genes cloned on a plasmid, and obtained the production of higher branched-chain alcohols with a titer of over 1.4 g/L in an MES system [26]. Besides that, the lactate dehydrogenase gene (*ldh*) is another commonly used integration site, whose deletion will reduce the formation of lactate especially under restricted oxygen conditions. Voss et al. integrated *cphA* (cyanophycin synthetase) into the *ldh* site for the biosynthesis of cyanophycin [52]. Similarly, Lutte et al. integrated *cphA* into the *norR2A2B2* site [49], encoding NO reductase, an enzyme involved in the denitrification pathway [67, 68]. Currently, genome integration has not been used as commonly as episomal plasmids due to the low gene dosage, limited integration sites, and lack of efficient integration tools. Although multiple-copy gene integration of P_*phaP*_-*OPH* done by Srinivasan et al. showed high stability, the production was still lower than that of the plasmid-based system [54]. Nevertheless, with the development of synthetic biology, genome integration, as a complementary approach to plasmid, will play increasingly important roles in biotechnological applications. Thus, it is expected that genome integration tools and more stable integration sites will be established and characterized in the near future, which will be further discussed in the "Genome editing" Section.

## Heterologous gene expression elements

A precise expression system, consisting of constitutive or inducible promoters, 5′-untranslated regions (UTRs), signal peptides, target genes, and terminators, is essential for constructing cell factories. To balance cell growth and product biosynthesis, a well-tuned expression system is highly desirable, which mainly depends on diverse elements determining transcription efficiency (e.g., promoters and terminators) and translation efficiency (e.g., ribosomal binding sites, RBS). In this section, these available elements in *C. necator* will be summarized.

### Constitutive and inducible promoters

In recent years, numerous well-characterized and controllable promoters have been developed, facilitating the applications in PHB synthesis and CO_2_ conversion. Native constitutive promoters related to PHB synthesis (P_*phaC1*_), pyruvate metabolism (P_*pdhE*_) [69], acetyl-CoA synthesis (P_*acoE*_) [70], and translation (P_*rrsC*_) [71] are employed in metabolic engineering. Among them, P_*phaC1*_ is the most commonly used [26, 59]. Unfortunately, these native promoters are relatively weak. Promoters derived from other organisms can be used to extend the promoter strength range. Notably, P_*lac*_ and its derivatives from *E. coli* can work as constitutive promoters in *C. necator* due to the absence of *lacI* and *lacY* homologs in the genome. Fukui et al. showed P_*tac*_ exhibited 1.5- to 2-fold higher GFP expression than that of P_*phaC1*_ [72]. Arikawa et al. compared the strength of various promoters (P_*trc*_ ≥ P_*lacUV5*_ > P_*trp*_) and P_*trc*_ was at least 20-fold higher than P_*phaC1*_ [73]. Although the absolute value may vary in different laboratories due to subtle differences in promoter architecture, cultivation conditions, and analytical devices, P_*trc*_ and P_*tac*_ (a mutant of P_*trc*_ that can initiate transcription more efficiently without the presence of catabolite activator protein, CAP) are relatively strong promoters in *C. necator*. Subsequently, Gruber et al. tested a series of promoters derived from bacteriophage T5 and P_*j5*_ was identified as the strongest promoter, about fivefold higher than P_*tac*_ [46]. To get promoters with a broad activity range, Li et al. constructed a P_*phaC1*_ promoter library by mutating the last four nucleotides of the -35 region, and obtained a variety of promoters weaker than the native P_*phaC1*_ [71]. Alagesan et al. combined core sequences of previously characterized promoters with upstream and downstream insulation sequences to construct a promoter library, in which four promoter variants were identified to be stronger than P_*j5*_ [74]. Johnson et al. adopted rational engineering approaches of point mutation, length alteration, incorporation of regulatory genetic element, promoter hybridization, and configuration alteration, and obtained a 42-promoter library displaying a wide range of activities based on P_*phaC1*_, P_*rrsC*_, P_*j5*_, and P_*g25*_ [75].

For more precise regulation, inducible promoters are preferred, particularly those derived from other organisms and synthetic systems, because of their orthogonality to *C. necator* and efficiency for directing carbon flux to the biosynthesis of the target products. AraC/P_*araBAD*_ from *E. coli* is the most widely used and exhibits the strongest transcription activity. Nonetheless, leaky expression and growth defects limit its further application [76, 77]. RhaRS/P_*rhaBAD*_, also from *E. coli*, seems to be a more promising regulatory system. Although the maximum induction level of RhaRS/P_*rhaBAD*_ is slightly lower than that of AraC/P_*araBAD*_, the induction ratio is much higher due to lower leaky expression [74]. Sydow et al. showed that the growth of *C. necator* was virtually not altered even at the highest expression level (i.e., 11 mM l-rhamnose) [61]. Modified LacI/P_*lac*_ system can be induced by isopropyl β-d-1-thiogalactopyranoside (IPTG) when lactose permease (*lacY*) was co-expressed. Bi et al. constructed pYIUV5Trfp, containing *lacY* and *lacI*, which was found to be functional in *C. necator* although its expression level was relatively low [47]. Hanko et al. demonstrated that MmsR/P_*mmsA*_ and HpdR/P_*hpdH*_ derived from *Pseudomonas putida* were highly inducible systems with 3-hydroxypropionic acid as both the inducer and carbon source [78]. PM/P_*xyls*_-m-toluic acid [47], AcuR/P_*acuRI*_-acrylate [74], and YpItcR/P_*ccl*_-itaconate [79] systems were found to work in *C. necator* as well in spite of high background expression levels. Synthetic gene circuits, which combine those characterized elements, can be constructed to further develop subtler inducible expression systems. Gruber et al. combined constitutive promoter P_*j5*_ with lac and cumate regulatory elements to construct LacI/P_*j5-lac*_ and CymR/P_*j5-cmt*_. CymR/P_*j5-cmt*_ was determined to be better because of lower leaky expression, slighter growth defects, and cheaper inducer [65]. Li et al. developed a synthetic anhydrotetracycline-controllable gene expression system TetR/P_*rrsC-tetO*_ by stepwise optimization of the type of *tetO* (*tetO1* and *tetO2*), the copy number of *tetO1*, and the expression level of *tetR* [71]. Similarly, Aboulnaga et al. constructed a TetR/P_*tolC-tetO*_ system with a high induction ratio [80]. Barnard et al. integrated T7 RNA polymerase gene under the control of P_*phaP*_ and used T7 promoter to achieve high-level recombinant protein expression just like pET system, the most widely used expression system in *E. coli* [81]. Bi et al. adopted a similar strategy to couple AraC/P_*araBAD*_ with T7 system [82]. Inducible promoters developed in recent years are summarized in Table 3, many of which are found to be orthogonal to each other. For instance, the addition of L-arabinose has no impact on RhaRS/P_*rhaBAD*_ and HpdR/P_*hpdH*_ [78].

**Table 3:** Comparison of inducible promoters developed in *C. necator*

Promoter	Inducer and concentration	Induction ratio	Effects on growth
AraC/P_*araBAD*_	10 mM L-arabinose	1200	Inhibited cell growth; not consumed
RhaRS/P_*rhaBAD*_	11 mM L-rhamnose	1960	Virtually not altered cell growth; not consumed
LacI/P_*lac*_	1 mM IPTG	~ 300	Inhibited cell growth at high concentration; not consumed
PM/P_*xyls*_	1 mM m-toluic acid	~ 300	–
AcuR/P_*acuRI*_	5 mM acrylate	33	Consumed
YpItcR/P_*ccl*_	5 mM itaconate	105	Not consumed
MmsR/P_*mmsA*_	10 mM 3-hydroxypropionic acid	51.5	Growth-retarding effect at the beginning of cultivation; consumed
HpdR/P_*hpdH*_	10 mM 3-hydroxypropionic acid	516.6	Growth-retarding effect at the beginning of cultivation; consumed
LacI/P_*j5-lac*_	1 mM IPTG	~ 7	Growth inhibition at high concentration; not consumed
CymR/P_*j5-cmt*_	120 μM p-cumate	22	Nearly no effect on growth
TetR/P_*rrsC-tetO*_	200 ng/mL anhydrotetracycline	~ 1100	No negative effect on growth; not consumed; antibiotic
TetR/P_*tolC-tetO*_	25 ng/mL doxycycline	~ 180	No negative effect on growth; not consumed; antibiotic

The induction ratio is recalculated based on AraC/P_*araBAD*_ as a benchmark according to references [47, 65, 71, 74, 78–80]

Although inducible promoters are effective for precise control of gene expression levels, inducer implementation significantly increases the cost of the whole bioprocess and limits their practical applications. On the contrary, some native promoters related to essential metabolism can implement auto-induction without the need of inducers. P_*phaP*_ is activated under phosphate limitation conditions, tightly coupling with PHB biosynthesis [53, 72, 83]. P_*cbbL*_ is induced under chemolithoautotrophic conditions and repressed on pyruvate and fructose [49, 84–86]. P_*acoD*_ and P_*acoX*_, related to the acetoin metabolism, are induced by acetoin and commonly used for metabolic engineering, although these two native promoters are rather weak [69, 87]. P_*SH*_ and P_*MBH*_ (hydrogenase promoters) are probably the strongest native ones identified so far in *C. necator*, which are induced on glycerol and repressed on fructose [88–90]. However, applications of these auto-inducible promoters, such as dynamic control, have not been fully explored yet. Overall, to deal with complex metabolic engineering tasks, more controllable and elaborate promoter systems should be constructed and tested. In addition to classical promoters, riboswitches that can precisely control the translation initiation rate can be explored in *C. necator* [91, 92].

### 5′-Untranslated regions (UTRs)

Translation efficiency mainly depends on the stability of mRNA and its ability to bind to ribosomes. Thus, the key to efficient translation is a suitable RBS, regardless of whether it is native, derived from *E. coli* or synthetically designed. Based on an RBS calculator developed by Voigt group [93], Alagesan et al. built an RBS library in *C. necator* with variable strengths, exhibiting more than a tenfold dynamic range [74]. They further verified that RBS strength could vary dramatically relying on the sequences of the promoters and target genes [94]. Therefore, in order to build a well-tuned expression system, a range of RBS should be individually evaluated in *C. necator*. In addition, T7 stem–loop structures and A/U-rich sequences can be employed to increase the stability of mRNA. Bi et al. added a T7 stem–loop structure between P_*araBAD*_ and RBS sequence, resulting in a twofold increase in the expression of RFP [47]. Alagesan et al. further demonstrated that T7 stem–loop structures could universally enhance gene expression in *C. necator*, while A/U-rich sequences could alter and fine-tune gene expression levels [74].

### Signal peptides

Signal peptides are not always needed, but can play crucial roles in some cases, such as the production of recombinant proteins. Secretion of target proteins into the culture broth or periplasmic space will not only save the cost of product recovery and purification, but also promote correct folding of the target proteins due to the specific oxidation–deoxidation environment. For instance, the signal peptide (i.e., a Sec signal peptide) of Caa, the periplasmic carbonic anhydrase, has the capability of secreting proteins to the periplasm [95]. Membrane-bound [NiFe]-hydrogenase (MBH) attaches to the plasma membrane and functions at periplasm, whose signal peptide (i.e., a Tat signal peptide) is located at the N-terminus of the small subunit HoxK [87]. Such signal peptides will be valuable for enzyme relocation for the assembly of complex metabolic networks. Recently, Tang et al. relocated VHb, an oxygen carrier, to the periplasm by the traction of MBH signal peptide to promote cell growth and PHB synthesis under oxygen-limiting conditions [96]. Unfortunately, only a limited number of signal peptides in *C. necator* have been characterized for synthetic biology applications and yet to be explored in the near future.

Besides the secretion of the target proteins, signal peptides can be employed for protein immobilization or pathway compartmentalization, by taking advantage of the accumulation of PHB granules in *C. necator*. The PHB-associated proteins such as PHB synthases (PhaC) [97], PHB depolymerases (PhaZ), and phasins (PhaP) [98], which are attached to the surface of PHB particles, can be used as anchor proteins. Barnard et al. employed fusion expression with phasins (PhaP) to attach the target proteins to the granules of PHB, leading to the formation of an “affinity matrix” in *C. necator* and simplified downstream purification steps [98]. Such application has not been fully explored in *C. necator*, but widely used in *E. coli* [99, 100]. Wong et al. constructed modular polyhydroxyalkanoate scaffolds for protein immobilization by fusing SpyCatcher protein with PHB synthases (PhaC) and fusing SpyTag with the target proteins [100]. A similar protein immobilization strategy can be adopted in *C. necator*, and it is expected that the co-location of multiple proteins on the PHB scaffolds (pathway compartmentalization) can be established in the near future for metabolic engineering applications [101].

### Target genes

Either for efficient production of foreign proteins or successful construction of long metabolic pathways, selection of endogenous or exogenous genes from suitable organisms is important. Codon optimization is an effective strategy for efficient expression of heterologous genes, because the GC content of *C. necator* genome is about 66% and much higher than that of most organisms [36, 102, 103]. Differences in GC content may impair transcriptional and translational efficiencies, limiting the enzyme activities and thus production yields. Grousseau et al. performed codon optimization of *adc* and *adh* from *Clostridium* species (about 30% GC), resulting in an 8.9 ± 3.0-fold increase in isopropanol production [103]. Another strategy for improving enzyme activities is to increase the copy number of the rate-limiting enzyme encoding genes, which is quite useful in breaking the “bottleneck” of long pathway. In the same case of isopropanol production, a second copy of *adh* increased the yield for additional 1.20 ± 0.18-fold [103].

### Terminators

A transcriptional terminator is important for avoiding massive energy waste for the production of unnecessary transcripts and the formation of undesirable secondary structures in a few cases [104]. However, the contribution of terminators to recombinant protein production and metabolic engineering applications is largely overlooked. To date, available terminators in *C. necator* are still rather limited, including pTOPO Terminator [54], *rrnB* T1 Terminator [105], *rrnD* T1 Terminator, and T7Te Terminator [80]. The *E. coli* derived *rrnB* T1 Terminator is the most commonly used [105]. Bi et al. used a dual-terminator (*rrnB* T1 + T7Te Terminator) to ensure transcription termination [47]. Aboulnaga et al. compared the bi-direction T7 Terminator with mini-*rrnD* T1 Terminator and found the latter to have a better termination efficiency [80].

## Platform strain and genome engineering

In order to construct robust and efficient microbial cell factories, platform strains should be modified via genome editing tools. In recent years, several *C. necator* hosts, such as for cultivating in an MES system or producing fatty acid derivatives, have been established. Limited by genome editing tools and basic metabolic knowledge of *C. necator*, adaptive evolution offers a powerful alternative for chassis engineering.

### Genome editing

**Fig. 2:**
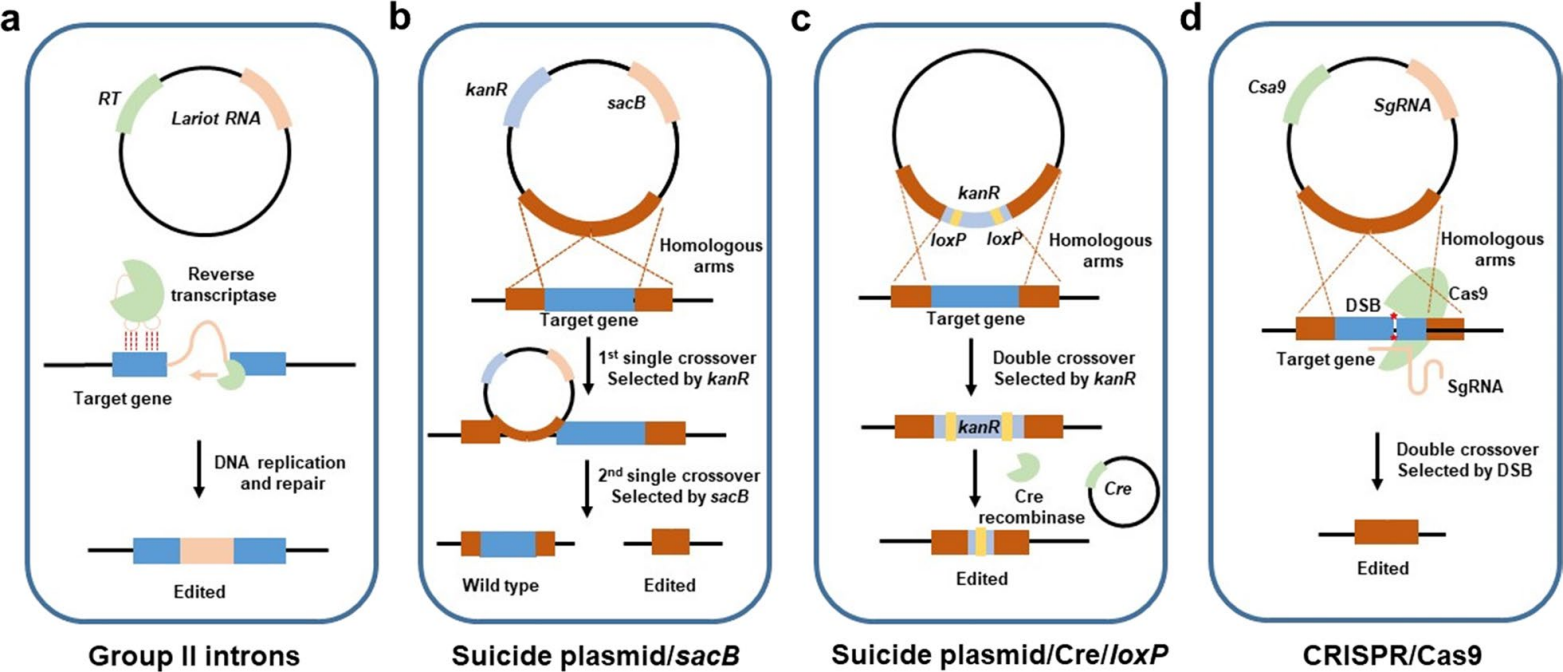
Genome editing tools developed in *C. necator* H16. **a** Target gene deletion via group II introns. **b** Target gene deletion via two rounds of single-crossover using *kanR* and *sacB* as selection and counter-selection markers, respectively. **c** Target gene deletion via double-crossover using *kanR* as a selection marker and maker recycling by the Cre/*loxP* system. **d** Target gene deletion via CRISPR/Cas9. DSB: double-strand break

Before the advent of new genome editing tools, UV mutagenesis [106] and chemical mutagenesis [107] played a crucial role in constructing many *C. necator* mutants still in use today. Tn5 transposon is another powerful tool in *C. necator* for random integration into the chromosome and it is superior to previous methods as it mainly causes single-gene mutations [108]. Peoples et al. constructed a PHB-negative mutant based on the insertion of Tn5 into the *phaC* site [109]. Barnard et al. achieved the integration of *OPH* expression cassette via Tn5 transposon [81]. Different from random integration based gene disruption, Park et al. developed a targeted gene knock-out system RalsTron, based on the group II introns. The functional genes were disrupted by inserting introns into specific gene loci, based on a mechanism named retrohoming (Fig. 2a) [110].

Homologous recombination (HR) is the most commonly employed strategy for gene disruption and insertion. Due to the relatively low HR efficiency, suicide plasmids have been constructed to greatly promote genome editing of *C. necator*, including pKNOCK (R6K ORI) [111], pLO1 (ColEl ORI) [112], pJQ200mp18 (P15A ORI) [113], and pK18mobsacB (pMB1 ORI) [114]. As their ORIs cannot replicate in bacteria other than enterobacteria, they function as suicide plasmids in *C. necator* and integrate into the chromosome via HR. Counter-selectable marker *sacB* (Table 2) and Cre/*loxP* system are employed to recycle the resistance marker for multi-round operation. As *sacB* (from *Bacillus subtilis*) encodes levansucrase that catalyzes the conversion of sucrose to levan, a cytotoxin, sucrose-containing medium can be used to select plasmid backbone free cells through a second-round single-crossover (Fig. 2b) [29, 113, 114]. Cre (from phage P1) is a kind of site-specific recombinase, specifically recognizing *loxP* sites and promoting HR between two *loxP* sites [115]. Gruber et al. integrated *lacY* into the *phaC* site by double-crossover and removed the chloramphenicol resistance marker using the Cre/*loxP* system (Fig. 2c) [65].

However, these tools suffer from low efficiency and being time-consuming. Knockout efficiency using RalsTron was only 12.5% in the study of Park et al. [110]. Although suicide plasmid-based system can perform knock-out and knock-in, two rounds of operation are required and the efficiency is still not satisfactory. The emergence of CRISPR/Cas technique provides more effective and time-saving tools. A single guide RNA (sgRNA) guides the Cas protein to bind and cut DNA specifically, resulting in the formation of a double-strand break (DSB), which is repaired by non-homologous recombination end joining (NHEJ) or HR to achieve gene knock-out or knock-in [116]. Xiong et al. was the first to apply CRISPR/Cas9 in *C. necator* and assembled three elements, sgRNA, Cas9, and donor DNA in one plasmid with editing efficiencies ranging from 78.3 to 100% (Fig. 2d) [117]. Due to the difficulties in transforming large plasmids to *C. necator*, the integration of large DNA fragments based on the CRISPR/Cas9 system has not been achieved yet. For synthetic biology applications of *C. necator*, more powerful genome engineering tools, e.g., multiplex genome editing technique either based on CRISPR/Cas9 or base editor [118, 119], should be developed in the near future.

### Adaptive evolution

Adaptive evolution is a powerful tool to engineer *C. necator* with complex phenotypes, such as high tolerance or high utilization rate of carbon sources, due to our limited knowledge of the metabolic and regulatory network. With the development of sequencing and omics technology, an in-depth understanding of genotype and phenotype relationships becomes possible. Liu et al. built a water-splitting biosynthetic system with the need for ROS (reactive oxygen species)-resistant variants of *C. necator*. *C. necator* was exposed to the water-splitting system for 11 consecutive days and sequencing analysis showed that mutations in the membrane protein related to cation/multidrug efflux system and transcriptional regulator contributed to ROS resistance [10]. Gonzalez-Villanueva et al. obtained *C. necator* 16 variant v6C6 with a specific growth rate in glycerol 9.5 times faster than the wild-type strain via adaptive laboratory evolution, and identified glycerol kinase as the key enzyme for improved glycerol utilization [120]. Claassens et al. combined short-term evolution and rational engineering, achieving CO_2_ assimilation via more efficient reductive glycine pathway other than the endogenous CBB cycle [59]. With the aid of synthetic biology tools, the combination of rational design and laboratory adaptive evolution is expected to play an increasingly important role in the construction and optimization of *C. necator* cell factories in the near future.

### Commonly used *C. necator* platform strains

The most commonly used *C. necator* host is the PHB-negative mutant (Table 4), in which carbon flow can be easily redirected to the synthesis of the target products. The first such mutant (H16PHB^−^4) was constructed by Schlegel et al. through 1-nitroso-3-nitro-l-methylguanidine (NMG) treatment [107]. Unfortunately, due to random mutagenesis, defects in fatty acid metabolism and regulation of CBB cycle were observed in H16PHB^−^4 [43]. Subsequently, H16Δ*phaCAB* and H16Δ*phaC1* were precisely constructed via targeted genome editing tools [83, 121]. In spite of the presence of multiple orthologues, the deletion of the main operon *phaCAB* or the key synthase gene *phaC1* nearly abolishes the capability of PHB biosynthesis in *C. necator*.

**Table 4:** Commonly used *C. necator* strains for different applications

*C. necator* strain	Genome description	Application	Reference
H16PHB^−^4	Chemical mutates	Value-added products	[107]
H16Δ*phaCAB*	Precisely constructed to block PHB synthesis	Value-added products	[121]
H16Δ*phaC1*	Precisely constructed to block PHB synthesis	Value-added products	[83]
H16 G^+^1	UV mutates	Able to use glucose	[106]
C5	H16Δ*H16_A0006* Δ*H16_A0008-9*	Improved electroporation efficiency	[117]
Re2303	H16Δ(*H16_A0459-0464*, *H16_A1526-1531*); Mutant deficient in native β-oxidation	Fatty-acid derived fuels and chemicals	[124]
BC4	Mutant in *acrC1*, *Hfq*, *H16_A2360*, and *H16_B0214*	ROS-tolerant strain to be applied in MES	[10]
v6C6	Mutant in *H16_A0689*, *H16_A1373*, *H16_A2507*, and *H16_A3075*	Improved glycerol utilization	[120]

Another effort was devoted to reversing the inability to utilize glucose. Schlegel et al. constructed H16 G^+^1 via UV mutagenesis [106] and Raberg et al. subsequently obtained a glucose-utilizing mutant (H16Δ*nagRnagE*Ala153Thr) via rational engineering [122, 123]. In recent years, more platform strains have been constructed to cater to the metabolic engineering needs for the biosynthesis of different products. For instance, Brigham et al. deleted two native β-oxidation operons in *C. necator*, which is suitable for the production of fatty acid-derived fuels and chemicals [124]. Besides these driven by direct metabolic engineering applications, platform strains for more efficient genetic manipulation have been established as well. Xiong et al. constructed a platform strain, C5, for electroporation by deleting putative restriction modification (RM) genes *H16_A0006* and *H16_A0008-9* [117].

## Transformation methods

Regardless of the expression of recombinant proteins, construction of exogenous pathways, or genome editing of *C. necator*, foreign DNA should be introduced into the host cell first. Thus, DNA transformation is a fundamental technique for synthetic biology. The transformation efficiency of non-model Gram-negative bacteria is usually low probably due to the complicated cell envelope structures. Thus, heat-shock-based chemical transformation is not feasible and conjugation and electroporation are commonly employed in *C. necator*. The efficiency of conjugation and electroporation is related to plasmid size and stability, transformation condition, and the host [125]. Notably, Sato et al. showed that the transformation efficiency of plasmid with the maintenance element parABS28 derived from pMOL28 was 500-fold higher [48].

### Conjugation

Conjugation is the process of transferring genetic materials between two bacteria via cell mating, whose advantage lies in generality, i.e., it is not affected by the endogenous RM systems of hosts [45]. Thus, foreign DNAs can be introduced to *C. necator* via: (1) introducing DNA to a donor strain (the most commonly used one is *E. coli* S17-1) via chemical transformation or electroporation; (2) introducing DNA from S17-1 to *C. necator* via cell mating. This process requires two essential elements: a transfer gene (*tra*) and a mobilization site (*mob*) including the origin of transfer (*oriT*) [45]. Commonly used plasmids in *C. necator* all have their own mobilization sequences. Gruber et al. showed that the mobilization efficiency of RP4 was about 10- and 50,000-fold higher than that of RSF1010 and pBBR1, respectively. The reason may lie in the donor strain S17-1, whose chromosome is integrated with the natural RP4 transfer sequences [126]. Mobilization sequences with high similarity to RP4 *mob* may perform interact better with RP4 transfer sequences and result in higher transformation efficiency [46]. Despite a high efficiency, conjugation requires a long experiment period and intensive labor with two rounds of cell culture. Therefore, a simple and time-saving method is preferred.

### Electroporation

Electroporation is a more direct method, introducing foreign DNA into the recipient strain in a single step. However, the efficiency was as low as 10^2^ ~ 10^3^ cfu/μg DNA depending on the plasmid size originally [127, 128]. Thus, two strategies have been employed to improve the transformation efficiency. Tee et al. systematically optimized the electroporation parameters, including transformation buffers, chemical treatment, electroporation voltage, cell concentration, and cell growth phase. The optimized electroporation protocol with cells grown to OD_600_ 0.6, a 15 min incubation in 50 mM CaCl_2_, two cell washes with glycerol, resuspension in 0.2 M sucrose, and 2.3 kV electroporation, resulted in a transformation efficiency of (3.86 ± 0.29) × 10^5^ cfu/μg DNA [125]. Xiong et al. found that the electroporation efficiency was limited by the endogenous RM systems and the disruption of the putative RM genes *H16_A0006* and *H16_A0008-9* in *C. necator* increased the electroporation efficiency more than 10^3^ times [117]. These efforts have made electroporation feasible even for some large-size plasmids.

### Chemico-physical transformation

Although common chemical transformation cannot get high enough transformation efficiency for *C. necator*, the combination with physical transformation (e.g., needle-like materials) makes it possible. Ren et al. tested the combination of five different chemicals (RbCl, lithium acetate, cesium chloride, dimethyl sulfoxide, and magnesium chloride) and four different nanomaterials (sepiolite, gold(III) chloride, multi-walled carbon nanotube, and chitosan) and found that the highest efficiency was obtained when cells were treated with gold(III) chloride and 0.1 M RbCl (3.49 × 10^4^ CFU/μg of pBBR1MCS2). Although the transformation efficiency is slightly lower than electroporation (under optimal conditions), it is much simpler and no special equipment is required [129].

## Synthetic biology applications of *C. necator* H16

With the development of genetic tools mentioned above, the use of *C. necator* as a biofuel-producing organism has become a hot spot [130]. Biofuel products that have been reported include alcohols (e.g., ethanol and isopropanol), fatty acids, ketones, alkanes, and terpenoids [131]. The general metabolic engineering strategy is to increase heterologous pathway activity and reduce the metabolic flux of competing pathways in *C. necator*. This section discusses the efforts that have been devoted to engineering *C. necator* with those above-mentioned genetic tools to synthesize biofuels and chemicals from CO_2_. Notably, *C. necator* has been engineered to synthesize a wide variety of chemicals from organic sources, such as fructose and fatty acids, which is not the major focus of and not included in this review. The products synthesized in *C. necator* from CO_2_ are listed in Table 5.

**Table 5:** Products synthesized in *C. necator* from CO_2_

Products	Vectors	Expression elements	Host	Fermentation conditions and titers (yields)	References
Acetoin	Plasmid (*Tc*^*R*^, RSF1010, *par*)	P_*phb*_-*alsSD*	H16Δ*acoABC*Δ*phaC1*Δ*phaC2*	Gas fermentation 0.32 mol acetoin/mol CO_2_	[188]
2,3-Butanediol	Integration (*phaC1AB1*)	P_*araBAD*_-*alsSD*-*sadh*	H16Δ*acoXABC* Δ*phaC1AB1*	Gas fermentation 32.0 ± 0.1 g/L	[139]
Isopropanol	Integration (*phaC1*)	P_*araBAD*_-*ctfAB*-*adc*-*sadh*-*phaA*	H16Δ*phaC1B1*	Gas fermentation 7.7 ± 0.2 g/L	[139]
	Plasmid pEG7b (*Kan*^*R*^, pBBR1)	P_*tac*_-*thl*-*ctf*-*adc*-*adh*	H16Δ*phaCAB*Δ*phaB2* Δ*phaB3*	Gas fermentation 3.5 g/L	[138]
	Plasmid pEG12 (*Kan*^*R*^, pBBR1)	P_*lac*_-*thl*-*thl*-*ctf*-*adc*-*adh*	H16Δ*phaCAB*Δ*phaB2* Δ*phaB3*	MES 216 mg/L	[25]
	Plasmid pEG12 (*Kan*^*R*^, pBBR1)	P_*lac*_-*thl*-*thl*-*ctf*-*adc*-*adh*	H16Δ*phaCAB*Δ*phaB2* Δ*phaB3*	MES ~ 600 mg/L	[10]
*n*-Butanol	Plasmid pLH205 (*Kan*^*R*^, pBHR1)	P_*CAT*_-*phaJ*-*phaAB*-*terOP*-*bldh*-*yqhD*	H16	Formic acid 30 mg/L	[151]
Isobutanol and 3-methyl-1-butanol	Plasmid JL26 (*Kan*^*R*^, pBBR1)	P_*lac*_-*ilvBHCD*-*kivd*	DJ21Δ*phaCAB*Δ*ilvE* Δ*bkdAB*Δ*aceE*	MES ~ 220 mg/L	[10]
	Integration (*phaB2C2*) plasmid pYL22 (*Kan*^*R*^, pBHR1)	int-P_*phaC1*_-*alsS*-*ilvCD* plasmid-P_*CAT*_-*kivd*-*yqhD*	H16Δ*phaCAB*	MES 140 mg/L	[26]
Fatty acids	Plasmids pCT (*Kan*^*R*^, pBBR1); pFP (*Cm*^*R*^, RP4)	pCT-P_*araBAD*_-*acc*-*LTes* pFP-P_*araBAD*_-*Fas*-*acpS*	H16Δ*phaC1*	Gas fermentation 60.64 mg/g CDW	[58]
Methyl ketones	Plasmid pJM20 (*Kan*^*R*^, pBBR1)	P_*araBAD*_-* ‘tesA*-*fadB*-*Mlut_11700*-*fadM*	Re2303Δ*phaCAB*	Gas fermentation 50 ~ 180 mg/L	[161]
Alka(e)ne	Plasmid pLC10 (*Kan*^*R*^, pBBR1)	P_*araBAD*_-*ado*-*aar*	H16Δ*phaCAB*	Gas fermentation 4.4 mg/L	[189]
α-Humulene	Plasmid pKR-hum (*Tc*^*R*^, RSF1010, *par*)	P_*rhaBAD*_-*zssI*-*erg20*-*hmgS*-*fni*-*hmgR*-*mvaK*-*mvaD*-*mvaK2*	H16PHB^−^4	MES 17 mg/g CDW	[19]
2-Hydroxy isobutyric acid	Plasmid pHCM (*Kan*^*R*^, pBBR1)	P_*lac*_-*meaB*-*hcmA*-*hcmB*	H16PHB^−^4	Gas fermentation 3.2 g/L	[190]
Trehalose	Plasmid pSEVA228 (*Kan*^*R*^, RP4)	P_*xyls*_-*setA*	H16	Gas fermentation 0.47 g/L	[191]
Sucrose	Plasmid pBADT (*Kan*^*R*^, pBBR1)	P_*araBAD*_-*SPS*-*SPP*-*scrY*	H16	Gas fermentation 180 mg/L	[192]
Lipochitooligosaccharides	Plasmid pBADT (*Kan*^*R*^, pBBR1)	P_*araBAD*_-*nodABC*	H16	Gas fermentation 1.4 mg/L	[192]
Lycopene	Plasmid (*Kan*^*R*^, pBBR1)	P_*lac*_-*CrtEBI*2	C5	MES 1.73 mg/L	[175]

DJ21is an isobutanol tolerant strain with constitutive alcohol dehydrogenase activity constructed by Jendrossek et al. [150]

### Alcohols

#### Isopropanol

Isopropanol (isopropyl alcohol or IPA) is mainly used as a solvent, but also as a feedstock to produce paints, cleaners, and cosmetic and chemical intermediates (such as esters and amines). The main driving force behind the development of fermentation production processes for pure IPA lies in the potential use as a precursor of isopropylene, which is one of the most important components of propylene in the chemical industry [132]. If the cost of IPA is low enough to serve as a biofuel molecule or a precursor of isopropylene, much larger markets can be expected [133]. Compared with the traditional chemical production method (e.g., by reducing acetone in the presence of excess hydrogen), microbial synthesis of IPA from renewable raw materials has great advantages, such as relatively mild conditions, no expensive catalysts required, and environmental friendly [134].

**Fig. 3:**
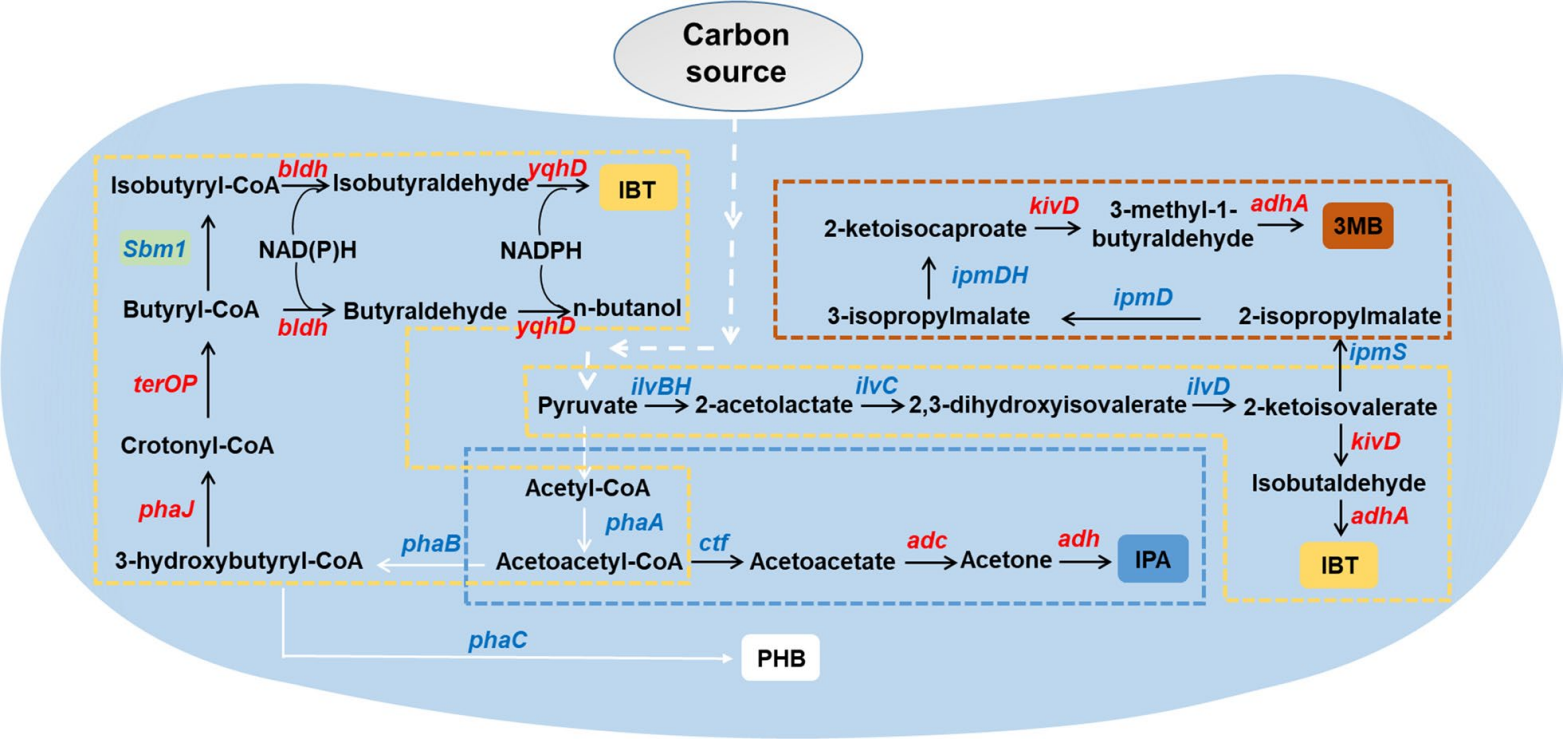
Schematic of the biosynthetic pathways for producing isopropanol, isobutanol, and 3-methyl-1-butanol in *C. necator* H16. The isopropanol pathway is shown in blue, two different isobutanol pathways in yellow, the 3-methyl-1-butanol pathway in orange, and the polyhydroxybutyrate pathway in white. Native genes are shown in blue, while heterologous genes are shown in red. IPA: isopropanol; IBT: isobutanol; 3MB: 3-methyl-1-butanol; PHB: polyhydroxybutyrate

*Cupriviadus necator* can produce large amount of PHB under unfavorable growth conditions such as nutrient limitation to store excess carbon. Thus, *C. necator* is an excellent host to produce IPA, which shares the same biosynthesis precursors, acetoacetyl-CoA, with PHB (Fig. 3). A few genetic modifications are sufficient to rewire the metabolic fluxes of the precursor from PHB accumulation to IPA production. The deletion of competing pathways, *phaB* and *phaC*, and the introduction of the heterologous genes from *Clostridium species* (*adc* and *adh*) resulted in successful production of IPA. Then codon optimization and increasing copy numbers of the pathway genes were employed to increase the heterologous gene expression levels. Furthermore, by using an inducible promoter P_*araBAD*_, the engineered strain Re2133/pEG7c produced IPA with a titer up to 3.44 g/L from fructose as a sole carbon source [103]. Nevertheless, the potential product toxicity is another issue that should be addressed. As previously reported, heat shock proteins, alcohol dehydrogenases, and efflux pump proteins have been shown to increase ethanol tolerance in a broad range of bacteria such as *C. acetobutylicum* and *E. coli* [135, 136]. Thus, Marc et al. overexpressed GroES and GroEL (heat shock protein family) to increase the stability of ADC and ADH, leading to increased IPA production. Finally, *C. necator* Re2133/pEG23 strain was able to produce 9.8 g/L IPA when fructose was used as the sole carbon source [137]. To achieve IPA production from CO_2_, Garrigues et al. designed a pressurized bioreactor to provide higher gas abundance and increase the gas transfer rate, with the IPA titer reaching as high as 3.5 g/L by gas fermentation [138]. Similarly, Bommareddy et al. established a continuous autotrophic fermentation system and obtained 7.7 g/L IPA [139]. Recently, an MES system was set up by coupling *C. necator* strain Re2133/pEG12 and water-splitting system, resulting in the production of 216 mg/L IPA [25]. Then, Liu et al. designed a novel electrode material to eliminate the generation of ROS and improved the titer of IPA to 600 mg/L with Co-P alloy cathode and CoPi anode [10]. Overall, great progress in IPA production from CO_2_ in *C. necator* has been made in recent years, whose titer was far beyond those by cyanobacteria under photosynthetic conditions, i.e., ~ 150 mg/L [140–142].

#### Isobutanol and 3-methyl-1-butanol

Compared with isopropanol, higher alcohols (e.g., *n*-butanol, isobutanol, and 3-methyl-1-butanol) have higher energy density and lower vapor pressure, hygroscopicity, and water solubility [143]. C4 alcohols have been found to be compatible with the current fuel distribution infrastructure of most countries, and can be used as fuels to run vehicles without any gasoline blending. In addition, isobutanol (IBT) is an important precursor for isobutene, which is widely used in refineries, rubber, and special chemical industries [144, 145].

The branched-chain amino acids catabolic pathway was engineered to produce fusel alcohols [146]. In this so-called Ehrlich pathway, branched-chain amino acids were converted to branched-chain α-keto acids by amino transferase, which were subsequently decarboxylated into the corresponding aldehydes and further reduced to fusel alcohols [147] (Fig. 3). Many microorganisms such as *B. subtilis, Saccharomyces cerevisiae,* and *Lactococcus lactis* were reported to produce IBT and 3-methyl-1-butanol (3MB) via the Ehrlich pathway [147–149]. While for *C. necator*, two additional enzymes (ketoisovalerate decarboxylase and alcohol dehydrogenase) should be introduced to produce IBT and 3MB [31]. A mutant strain of *C. necator* H16 with constitutive alcohol dehydrogenase activity and deficient PHB synthesis was chosen as the parent strain [150]. Subsequent overexpression of the heterologous *kivd* gene and the branched-chain amino acid biosynthesis pathway genes (*ilvBHCD*) resulted in the production of IBT and 3 MB. To further increase the production, other carbon sinks (i.e., valine-specific transaminase gene, a branched-chain keto acid dehydrogenase gene, and a pyruvate dehydrogenase gene) were deleted and the engineered strain produced 270 mg/L IBT and 40 mg/L 3 MB, respectively, when using fructose as the sole carbon source [121]. To enable IBT and 3 MB production from CO_2_, Liu et al. inoculated this engineered strain in MES as well and finally the total titers of IBT and 3MB reached up to ~ 220 mg/L [10]. Similarly, an IBT-producing strain LH74D constructed by Li et al. was cultivated in MES. To minimize the cytotoxicity of ROS, a porous ceramic cup was used to shield the anode and thus provide more chances for ROS quenching. Such a system resulted in the production of over 140 mg/L (total of IBT and 3 MB) biofuels with electricity and CO_2_ as the sole energy and carbon sources, respectively [26]. Notably, besides Ehrlich pathway, Black et al. designed a novel CoA-dependent pathway, constituted of chain elongation, rearrangement, and modification, for the synthesis of IBT. The endogenous isobutyryl-CoA mutase gene *Sbm1* was overexpressed to rearrange carbon flux from *n*-butanol to IBT and the engineered strain was able to produce 32 mg/L IBT with fructose as the sole carbon source [151] (Fig. 3). One of the factors limiting the ability of *C. necator* to produce IBT lies in low tolerance, i.e., lower than 0.5% (v/v). To address the product toxicity issues, Amanda and his colleagues constructed an IBT-tolerant strain by experimental evolution, which were able to grow in 2.5% (v/v) IBT and would be explored for IBT production in the near future [152]. Although the titer of IBT achieved in *C. necator* is far away from industrial application, the potential value of direct conversion of CO_2_ to IBT is still appealing. The titer of IBT in cyanobacteria has been increased to as high as ~ 1 g/L [147, 153, 154], indicating room for further engineering of *C. necator*.

### Fatty acids and derivatives

**Fig. 4:**
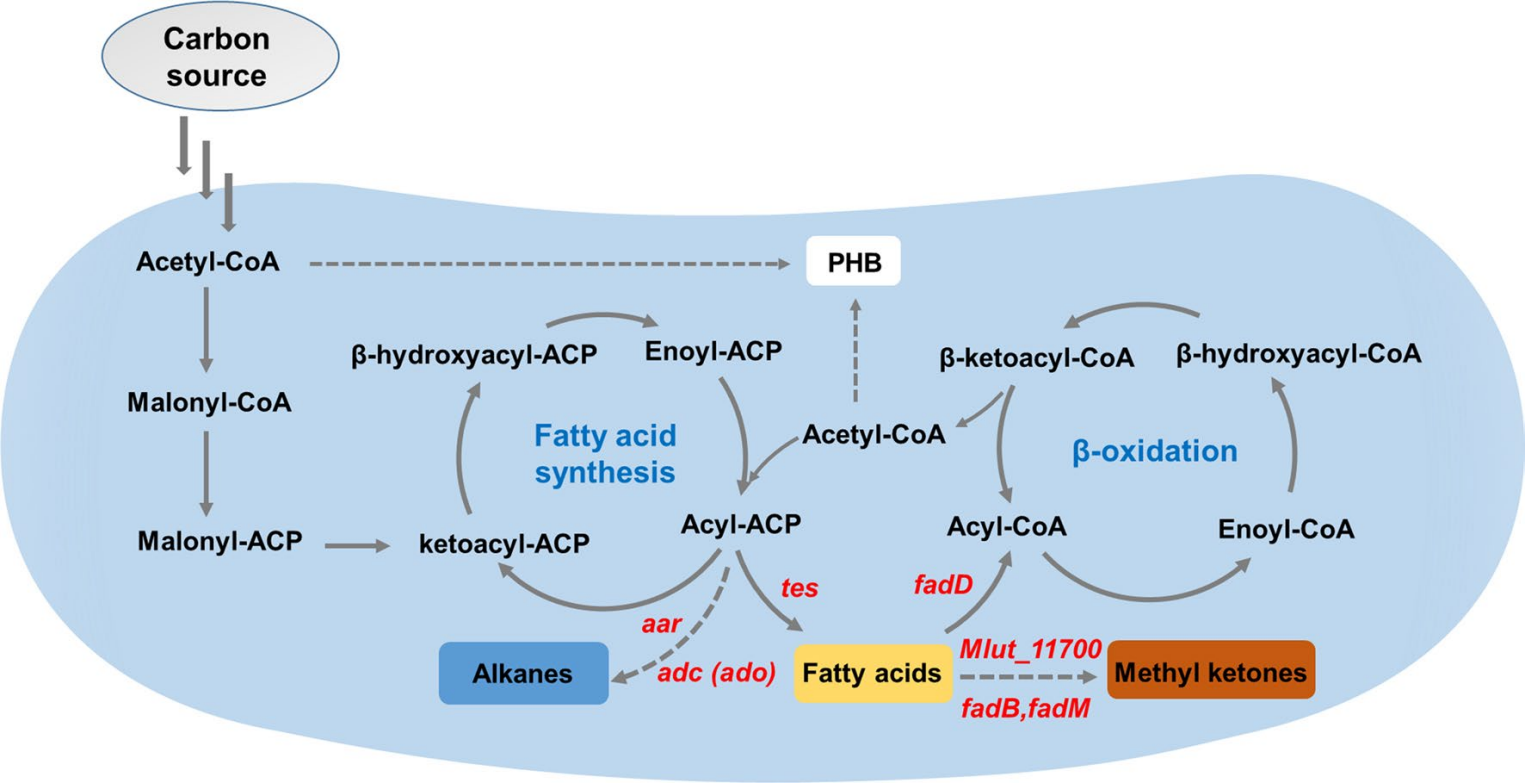
Schematic of the biosynthetic pathways for producing fatty acids and derivatives in *C. necator* H16. Main related pathways include fatty acid biosynthesis, β-oxidation cycle, and PHB synthesis, with acetyl-CoA as a central building block. Heterologous genes to synthesize fatty acids, alkanes, and methyl ketones are shown in red. PHB: polyhydroxybutyrate

Fatty acids, a large category of important chemicals, have been the focus of metabolic engineering and can be further converted into valuable biofuels, e.g., fatty acid methyl esters (FAMEs) [155]. During fatty acid biosynthesis, acetyl-CoA is iteratively condensed on an acyl carrier protein (ACP) scaffold. However, fatty acids can also be consumed through the β-oxidation pathway (Fig. 4) [124, 156, 157]. Over 50 homologues of β-oxidation enzymes have been identified in the genome of *C. necator,* which is much more than other model organisms such as *E. coli* [41]. Therefore, the synthesis of fatty acids and derivatives becomes a significant challenge in *C. necator*, which requires the block of β-oxidation pathway. Chen et al. overexpressed UcFatB2, which is a selective thioesterase for 12-carbon acyl-ACP substrates from the plant *Umbellularia californica*, in *C. necator* to produce laurate. The disruption of PHB synthesis and the acyl-CoA ligase gene *fadD3*, an entry point of fatty acids into β-oxidation, led to the production of total fatty acids increased up to 2.8-fold. Considering that laurate was still consumed in *C. necator*, three most highly upregulated acyl-CoA ligases were identified via RNA-Seq, the deletion of which resulted in the production of total fatty acids up to 62 mg/L [158]. Subsequently, Li et al. developed an autotrophic fermentation technique and obtained 60.64 mg/g CDW free fatty acids from CO_2_, using *C. necator* with pCT-P_*araBAD*_-*acc*-*LTes* and pFP-P_*araBAD*_-*Fas*-*acpS* [58].

Alkanes are the predominant constituents of gasoline, diesel, and jet fuels [159]. Alkanes, which are synthesized from acyl-ACP, have been produced in several microorganisms including cyanobacteria, bacteria, yeast, and fungi [160]. To produce alkanes in *C. necator*, acyl-ACP reductase (*aar*) and aldehyde decarbonylase (*adc*) encoding genes were overexpressed with the genetic toolbox developed by Bi et al. and the engineered *C. necator* strain produced 6 mg/L of total hydrocarbons [47]. Similarly, Crepin et al. introduced acyl-ACP reductase (*aar*) and an aldehyde deformylating oxygenase (*ado*) encoding genes to H16Δ*phaCAB*. Through codon, gene copy number, promoter, and RBS optimization, 435 mg/L of alkanes was produce from fructose and autotrophic alkane production was achieved. Although the autotrophic alkane production level was low (4.4 mg/L), it represented the first report to produce alka(e)nes from CO_2_ [161]. Furthermore, Crepin et al. increased alka(e)nes production up to 1.48 g/L (from fructose) by the expression of endogenous and heterologous ferredoxin–ferredoxin reductase systems [162].

Medium-chain methyl ketones, commonly found in microorganisms, plants, insects, and mammalian cells [163], have a variety of applications, such as pheromones, natural insecticides, flavoring in food, and diesel fuel blending agents [164, 165]. *C. necator* is capable of producing methyl ketones by modifying fatty acids metabolism under heterotrophic and autotrophic conditions. As fatty acids are the precursor of methyl ketones, Müller et al. overexpressed a cytoplasmic version of the TesA thioesterase in Re2303Δ*phaCAB*, whose production of free fatty acids was more than 150-fold higher than that of the wild type. Subsequently, three heterologous genes (acyl-CoA oxidase gene *Mlut_11700* from *Micrococcus luteus* and *fadB* and *fadM* from *E. coli*) were overexpressed in *C. necator*, and the finally engineered *C. necator* strain produced methyl ketones up to 50 ~ 65 mg/L under heterotrophic conditions and 50 ~ 180 mg/L under chemolithoautotrophic growth conditions, respectively [161].

### Isoprene and terpenes

**Fig. 5:**
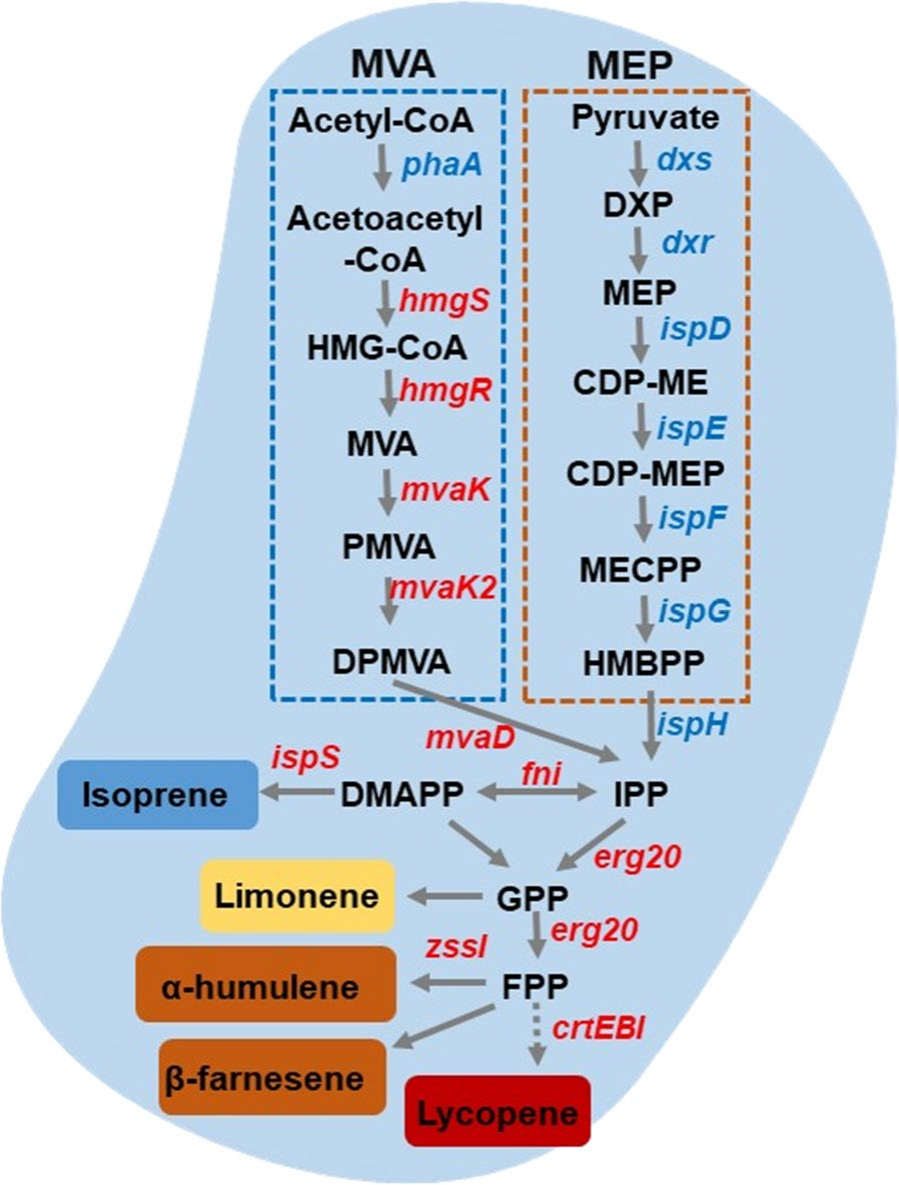
Schematic of the biosynthetic pathways for producing terpenoids in *C. necator* H16. Mevalonate (MVA, shown in blue) pathway and 2-C-methyl-D-erythritol-4-phosphate (MEP, shown in orange) pathway are two major pathways for terpenoid biosynthesis. Native genes are shown in blue, while heterologous genes are shown in red. HMG-CoA: 3-hydroxy-3-methyl glutaryl coenzyme A; MVA: mevalonic acid; PMVA: mevalonate-5-phosphate; DPMVA: mevalonate-5-pyrophosphate; DXP: 1-deoxy-D-xylulose-5-phosphate; MEP: 2-C-methyl-d-erythritol-4-phosphate; CDP-ME: 4-(cytidine 5′-diphospho)-2-C-methyl-d-erythrito; CDP-MEP: 4-(cytidine 5′-diphospho)-2-C-Methyl-d-erythritol-4-phosphate; MECPP: 2-C-methyl-d-erythritol-2,4-cyclodiphosphate; HMBPP: 1-hydroxy-2-meyhyl-2-butenyl-4-diphosphate; IPP: isopentenyl-5-pyrophosphate; DMAPP: dimethylallyl-pyrophosphate; GPP: geranyl-pyrophosphate; FPP: farnesyl-pyrophosphate

Terpenoids are widespread in the nature. More than 22,000 terpenoids have been reported, representing the largest group of natural products [166]. Traditionally, terpenes are used as the key fragrance compounds in perfumes and medicines. Furthermore, terpenes can serve as substitutes for chlorinated solvents in applications such as cleaning of electronic components. Because the physicochemical properties of terpenes are similar to petroleum-based fuels, another potential application was the use as substitutes for petroleum fuels [167, 168].There are two main synthetic pathways of terpenes in nature: the classic mevalonate (MVA) pathway and 2-C-methyl-d-erythritol-4-phosphate (MEP) pathway. The MVA pathway is widespread in eukaryotes, whereas the MEP pathway is prevalent in bacteria, e.g., *C. necator* H16. The MVA pathway converts acetyl-CoA to isopentenyl-5-pyrophosphate (IPP) and then an IPP isomerase maintains the balance between IPP and dimethylallyl-pyrophosphate (DMAPP). The MEP pathway consists of seven steps to convert glyceraldehyde-3-phosphate and pyruvate to IPP and DMAPP, which are building blocks for longer chain precursors of terpenes [169–171] (Fig. 5).

Isoprene (C5) is widely used in the synthetic rubber industry and fuel additives, whose precursor is DMAPP. Lee et al. introduced MVA genes and an isoprene synthase gene (*ispS*) to *C. necator* H16 and obtained 3.8 µg/L isoprene through codon and promoter optimization [102]. Monoterpenoids (C10), such as limonene, have strong fragrance and biological activity, and are important raw materials in pharmaceutical, food, and cosmetic industries. Jannson et al. achieved the production of limonene by chemolithoautotrophic culture of engineered *C. necator* [172]*.* Sesquiterpenes (C15), the largest subgroup of terpenoids, has a wide application in industry, such as β-farnesene, a precursor for a jet fuel additive, and α-humulene, a potential drug to treat cancer. Milker et al. obtained 26.3 ± 1.3 µM β-farnesene [173] and 2 g/L α-humulene [174] in a fed-batch mode on fructose as carbon source by expressing β-farnesene synthase and α-humulene synthase, respectively. Both studies showed that additional MVA expression contributed little to increase the titer of terpenoids, which might be resulted from the poor expression of hydroxymethylglutaryl-CoA reductase (*hmgR*). Furthermore, to produce terpenoids from CO_2_ directly, Krieg et al. inoculated H16PHB-4/pKR-hum in MES to produce α-humulene, which is the first report on chemolithoautotrophic production of a terpene. The titer of α-humulene reached 10 mg/g cell dry weight (CDW) under heterotrophic conditions and 17 mg/g CDW under chemolithoautotrophic conditions [19]. Recently, Wu et al. used the CO_2_ abundant real exhaust gas as the feedstock and achieved the production of 1.73 mg/L lycopene (C40), representing the most complex nonnative molecules in MES [175]. However, different with the production of diversified terpenes from CO_2_ in cyanobacteria [176, 177], the terpenes spectrum of *C. necator* is still rather limited, indicating a need for more extensive studies.

## Conclusions

This review summarizes genetic tools for *C. necator* from four perspectives: genetic engineering vectors, heterologous gene expression elements, platform strain and genome engineering, and transformation methods. Although many efforts have been devoted to expanding the toolkit, there remains an urgent need for more advanced methods, especially the CRISPR-based genome editing and gene regulation tools, e.g., CRISPRi [178] and base editor [179], to facilitate more complex metabolic engineering applications. Besides, computational simulation tools for *C. necator* are rather limited nowadays [180, 181]. To guide metabolic engineering, more in silico design tools, such as flux balance analysis (FBA) and elementary mode analysis (EMA), should be developed.

In addition, this review summarizes the value-added products converted from CO_2_ in *C. necator*, including alcohols, fatty acids, and terpenoids. Although CO_2_ valorization is mainly achieved via gas fermentation (H_2_, O_2_, and CO_2_), MES is a more promising method. Nevertheless, many challenges remain for wide applications of MES: (1) to improve the efficiency of CO_2_ fixation via metabolic engineering of CCB cycle or introducing new yet more efficient pathways; (2) to clarify and enhance the energy transfer process from the electrode to *C. necator*; (3) to diversify the product spectrum and increase the production yield; and (4) to better couple *C. necator* with the inorganic system.

In addition to CO_2_ conversion, *C. necator* has been expanded for applications in new areas. For example, *C. necator* was used for artificial selection and directed evolution studies of RuBisCO (ribulose 1,5-bisphosphate carboxylase/oxygenase, the rate-limiting enzyme for CO_2_ fixation) [182], the absorption and quantification of rare earth elements [183, 184], and hybrid photosynthesis [185–187]. With the development of more genetic engineering tools, the applications of *C. necator* are yet to be explored.

In summary, great progress has been made on genetic toolkit and synthetic biology applications of *C. necator*. Nevertheless, due to our limited knowledge of such a non-model microorganism, more efforts should be devoted to making *C. necator* as efficient cell factories for the conversion of CO_2_ to value-added products.

## Data Availability

Not applicable.

## Abbreviations

MES: Microbial electrosynthesis
ED: Entner–Doudoroff
CBB: Calvin–Benson–Basham
PHA: Polyhydroxyalkanoate
ORI: Origin of replication
MM: Minimal medium
PHB: Polyhydroxybutyrate
UTR: Untranslated region
CAP: Catabolite activator protein
IPTG: Isopropyl β-d-1-thiogalactopyranoside
MBH: Membrane-bound [NiFe]-hydrogenase
HR: Homologous recombination
sgRNA: Single guide RNA
DSB: Double-strand break
NHEJ: Non-homologous recombination end joining
ROS: Reactive oxygen species
NMG: 1-Nitroso-3-nitro-l-methylguanidine
RM: Restriction modification
IPA: Isopropanol
IBT: Isobutanol
3MB: 3-Methyl-1-butanol
FAMEs: Fatty acid methyl esters
ACP: Acyl carrier protein
MVA: Mevalonate
MEP: 2-C-methyl-d-erythritol-4-phosphate
IPP: Isopentenyl-5-pyrophosphate
DMAPP: Dimethylallyl-pyrophosphate
FPP: Farnesyl pyrophosphate
CDW: Cell dry weight
RuBisCO: Ribulose 1,5-bisphosphate carboxylase/oxygenase
FBA: Flux balance analysis
EMA: Elementary mode analysis
